# Metabolic detection of malignant brain gliomas through plasma lipidomic analysis and support vector machine-based machine learning

**DOI:** 10.1016/j.ebiom.2022.104097

**Published:** 2022-06-07

**Authors:** Juntuo Zhou, Nan Ji, Guangxi Wang, Yang Zhang, Huajie Song, Yuyao Yuan, Chunyuan Yang, Yan Jin, Zhe Zhang, Liwei Zhang, Yuxin Yin

**Affiliations:** a Institute of Precision Medicine, Peking University Shenzhen Hospital, Shenzhen 518036, China; b Institute of Systems Biomedicine, Department of Pathology, School of Basic Medical Sciences, Peking-Tsinghua Center for Life Sciences, Peking University Health Science Center, Beijing 100191, China; c Department of Neurosurgery, Beijing Tiantan Hospital, Capital Medical University, National Clinical Research Center for Neurological Diseases, Beijing 100070, China

**Keywords:** Malignant brain gliomas, Machine learning, SVM, Plasma biomarker, Lipidomics

## Abstract

**Background:**

Most malignant brain gliomas (MBGs) are associated with dismal outcomes, mainly due to their late diagnosis. Current diagnostic methods for MBGs are based on imaging and histological examination, which limits their early detection. Here, we aimed to identify reliable plasma lipid biomarkers for non-invasive diagnosis for MBGs.

**Methods:**

Untargeted lipidomic analysis was firstly performed using a discovery cohort (n=107). The data were processed by a support vector machine (SVM)-based discriminating model to retrieve a panel of candidate biomarkers. Then, a targeted quantification method was developed, and the SVM-based diagnostic model was constructed using a training cohort (n=750) and tested using a test cohort (n=225). Finally, the performance of the diagnostic model was further evaluated in an independent validation cohort (n=920) enrolled from multiple medical centers.

**Findings:**

A panel of 11 plasma lipids was identified as candidate biomarkers with an accuracy of 0.999. The diagnostic model developed achieved a high performance in distinguishing MBGs patients from normal controls with an area under the receiver-operating characteristic curve (AUC) of 0.9877 and 0.9869 in the training and test cohorts, respectively. In the validation cohort, the 11 lipid panel still achieved an accuracy of 0.9641 and an AUC of 0.9866.

**Interpretation:**

The present study demonstrates the applicability and robustness of utilizing a machine learning algorithm to analyze lipidomic data for efficient and reliable biomarker screening. The 11 lipid biomarkers show great potential for the non-invasive diagnosis of MBGs with high throughput.

**Funding:**

A full list of funding bodies that contributed to this study can be found in the Acknowledgments section.

**Research in context**

**Evidence before this study**

Computed tomography (CT) and magnetic resonance imaging (MRI) are routinely utilized for detection of malignant brain gliomas, but the cost, side effect of radiation and relative inaccessibility dampen their application. Tumor biopsy-based strategies, such as genetic profiling of specimens obtained by biopsy, or sequencing of circulating tumor DNA (ctDNA) from cerebrospinal fluid, are too invasive for repeated sampling. Liquid biopsy-based strategies have also been explored for the diagnosis of gliomas, such as detecting ctDNA in blood, but the sensitivity is not high enough for clinical practice. A novel non-invasive strategy based on highly accurate and sensitive biomarkers is still urgently needed.

**Added value of this study**

In the current study, we proposed a strategy combining lipidomics and an SVM-based machine learning algorithm to screen plasma biomarkers for malignant brain gliomas. Lipidomics analysis was firstly performed to explore lipid expression profile in plasma and then SVM modeling was performed to retrieve lipid markers. Finally, we established a targeted liquid chromatography-multiple reaction monitoring-mass spectrometry (LC-MRM-MS) assay method and an SVM-based diagnostic model using a panel of 11 lipids. The performance of this diagnostic model is evaluated in large cohorts deriving for different medical centers.

**Implications of all the available evidence**

Our findings reveal that the combination of lipidomics and machine learning algorithm is a powerful and efficient strategy for biomarker screening. The marker panel and diagnostic model we developed in this present study showed prospect in future clinical applications with advantages including non-invasive sample collection, quick analysis and high accuracy.

Alt-text: Unlabelled box

## Introduction

Malignant brain gliomas (MBGs) are the most common primary brain malignancies in adults. Increasing evidences have already suggested that early detection of cancer can be attributed to better prognosis or even cure in some malignancies.^1^^,^^2^ Meanwhile, nearly all the MBGs patients will suffer from recurrence after initial treatment. Therefore, improvement in accurate detection and monitoring of recurrence over time would remarkably benefit patients. Currently, computed tomography (CT) and magnetic resonance imaging (MRI) are routinely utilized for the initial detection and recurrence-monitoring of MBGs, but the cost, side effect of radiation and relative inaccessibility dampen their application in large-scale screening for patients. Besides, concerning that MBGs reside in brain, tumor biopsy-based strategies, such as genetic profiling of specimens obtained by fine needle aspiration biopsy,^3^ or sequencing of circulating tumor DNA (ctDNA) from cerebrospinal fluid (CSF),^4^ are too invasive for repeated sampling.

Recently, with the rapid development of second-generation sequencing and mass spectrometry, liquid biopsy-based methods have emerged as promising non-invasive diagnostic modalities for cancer diagnosis. Several kinds of biomolecules in blood have been found as potential biomarkers for cancers, such as ctDNA, RNA, antigen and proteins,^1^^,^^5^, ^6^, ^7^ demonstrating the applicability and potential of liquid biopsy in cancer diagnosis. Similar strategies have also been explored for the diagnosis of gliomas, such as detecting ctDNA, but the sensitivity of these methods is still not enough and its implementation into clinical practice remains a significant challenge.^8^ Therefore, a novel non-invasive strategy based on highly accurate and sensitive biomarkers is still urgently needed to facilitate diagnosis of MBGs in a clinical setting.

Lipidomics is a prevailing analytical strategy that targets the entire lipid species present in a given biological sample.^9^ Because of the important functions of lipids in critical biological processes such as energy metabolism,^10^ signal transduction and lipid membrane reconstruction,^11^ the study of dysregulated lipids in disease is a promising way to understand disease pathology or to screen for biomarkers. In general, the tight junctions between endothelial cells that form the blood brain barrier restrict penetration of hydrophilic molecules. However, the large surface area of the lipid membranes of the endothelium offers an effective diffusive route for lipid-soluble molecules.^12^^,^^13^ What's more, several published studies have reported potential blood lipid biomarkers for brain related diseases, such as neurodegenerative disease,^14^ traumatic brain injury^15^ and psychiatry.^16^ However, there are few lipidomic studies on brain gliomas and the potential of lipid markers for diagnosis remains relatively unexplored.

Machine learning (ML) refers to the use of data analysis to establish effective detection or verification models, and is an important part of artificial intelligence (AI).^17^ As the development of algorithms has advanced in recent years,^18^ ML approaches have begun to demonstrate their promise for broader applications.^19^^,^^20^ Support vector machines (SVM), as a classic member in ML, have also shown their advantages in medically related studies.^21^, ^22^, ^23^ In last few decades, the successful application of ML in clinical diagnosis of novel molecular markers, genetic alterations and protein changes, has been reported.^24^^,^^25^ Here in the present study, we propose a combination of lipidomics and an SVM-based ML algorithm as a powerful strategy for efficient and accurate biomarker screening for malignant brain gliomas. Based on an initial panel of candidate markers, we further established a targeted liquid chromatography-multiple reaction monitoring-mass spectrometry (LC-MRM-MS) assay method and a diagnostic model for convenient clinical applications.

## Methods

### Patients

Four sample cohorts (discovery, training, test and validation) comprising a total of 2,002 participants including MBGs patients (group name MBGs) and healthy controls (group name NC) were enrolled between June 2018 and September 2019. Baseline characteristics of the participants are summarized in Table 1. The inclusion criteria for MBGs patients were as follows: 1. without prior antitumor treatments; 2. prior tumor resection and histological diagnosis with MBGs according to the 2016 World Health Organization Classification of Central Nervous System Tumors;^26^ 3. availability of blood specimens for analyses and 4. completion of voluntary informed consent to participate in this study. NC groups were recruited from individuals undergoing annual physical examination from the physical examination center of hospitals under the following inclusion criteria: matched with the MBGs groups in terms of age and gender, and no history of cancer or systemic diseases. For the discovery cohort, plasma samples were collected at Tiantan Hospital, Beijing, China (MBGs group, n=72) and Peking University Third Hospital, Beijing, China (NC group, n=35). The training cohort of 750 participants was from Tiantan Hospital (MBGs group, n=385), Haidian Hospital, Beijing, China (NC group, n=173,) and Peking University Third Hospital (NC group, n=192). The test cohort of 225 participants was from Tiantan Hospital (MBGs group, n=115), Haidian Hospital (NC group, n=52) and Peking University Third Hospital (NC group, n=58). The validation cohort of 920 participants was composed of newly collected cases from May 2019 to September 2019 at Tiantan Hospital (MBGs group, n=351; NC group, n=61), Haidian Hospital (NC group, n=224) and Peking University Third Hospital (NC group, n=284).

**Table 1:** Characteristics of the subjects in each cohort.

	Discovery cohort (n=107)		Training cohort (n=750)		Test cohort (n=225)		Validation cohort (n=920)	
Variable	Brain gliomas	Healthy control	Brain gliomas	Healthy control	Brain gliomas	Healthy control	Brain gliomas	Healthy control
No. of cases	72	35	385	173+192	115	52+58	351	61+224+284
Age (year)	43±13.9	44±15.2	45±13.1	45±12.8	46±13.4	43±12.9	46±13	45±12.9
Sex (male/female)	42/30	20/15	229/156	171/194	73/42	54/56	196/155	283/286
Glioma grade								
WHO grade								
II								
A	15		45		20		76	
OA	8		67		15		44	
O	0		41		5		22	
III								
AOA	6		35		12		26	
AO	5		27		9		17	
AA	2		17		5		27	
IV								
GBM	36		153		49		139	

Data are presented as the mean ± SD. Abbreviations used are: A: astrocytoma; OA: oligoastrocytoma; O: oligodendroglioma; AOA: anaplatic oligoastrocytoma; AO: anaplatic oligodendroglioma; AA: anaplatic astrocytoma; GBM: glioblastoma.

### Plasma collection

For enrolled MBGs patients, 4 mL of arterial or peripheral blood was collected in tubes containing EDTA at the time of surgery. For NC group, peripheral blood was collected on the morning of the medical examination. All participants, including patients and healthy controls had fasted at least 8 hours before blood collection. Whole blood was centrifuged at 2500 g for 15 min and aliquots of supernatant (plasma) were transferred into cryovials and stored at -80°C.

### Chemicals

Ammonium acetate was purchased from Sigma-Aldrich (St. Louis, MO, USA). Formic acid, HPLC grade isopropanol (IPA), acetonitrile (ACN) and methanol were purchased from Fisher Scientific (USA). Deionized water was produced by a Milli-Q system. All standard chemicals were of analytical grade with typical purity of >99%. Phosphatidylcholine (PC 16:0-18:1, 16:0-18:2, 16:0-20:4, 16:0-22:6, 18:0-18:1, 18:0-18:2 and 18:0-20:4), lysophosphatidylcholine (LPC 13:0, 16:0 and 18:0) and triglyceride (TG 18:1-18:2-18:3) were purchased from Avanti Polar Lipids (USA).

### High-performance liquid chromatography and mass spectrometry

Lipids were extracted from plasma samples using liquid-liquid extraction. For untargeted lipidomics, 50 μl of plasma were mixed with 200 μl of chloroform/methanol (2:1, V/V). For targeted quantification, lipids were extracted from 25 μl of samples with 100 μl of chloroform/methanol (2:1, V/V) containing LPC 13:0 and glyceryl trioctanoate (TG 8:0-8:0-8:0) at 10 mg/ml as internal standards. For untargeted lipidomics, an Ultimate 3000 liquid chromatography coupled with a Q-Exactive MS was used in data dependent acquisition mode. Chromatographic separation was performed on a X-select CSH C18 column (4.6 mm × 100 mm, 2.5 μm). Two mobile phases were used for gradient elution: (A) ACN/water (3:2, V/V) and (B) IPA/ACN (9:1, V/V). Both mobile phase A and B contained 10 mM ammonium acetate and 0.1% formic acid. The gradient program was as follows: 0 min - 40% B; 0.5 min - 40% B; 0.6 min - 50% B; 6.6 min - 60% B; 6.7 min 75% B; 9.7 min – 99% B; 14 min - 99% B; 14.5 min - 40% B; and 19 min - 40% B. A pooled plasma sample was prepared as QC to assess the stability of the LC-MS instrument and ensure the reliability of the data. A QC sample was run before and after the sequence and after every 15 sample runs in the sequence. For targeted quantification, a Nexera UHPLC system coupled with a QTRAP 6500 MS was used in multiple reaction monitoring (MRM) mode. Chromatographic separation was performed on a X-select CSH C18 column (2.1 mm × 100 mm, 2.5 μm) using the same mobile phases as for the untargeted lipidomics. Optimized MRM parameters were included in Table S1.

### Lipid identification and quantification

The acquired lipidomic data (.raw) were processed using MS-DIAL software^27^ according to the instructions in the software tutorial. The MS/MS spectra-based lipid identification was performed in MS-DIAL by searching the acquired MS/MS spectra against the software's internal in silico MS/MS spectra database (LipidBlast database, version: LipidDBs-VS23-FiehnO). Chemical standards were used for identity validation for the 11 lipid markers. The acquired MRM data was processed by MultiQuant software (AB Sciex), areas of the XICs of targeted lipids were calculated and normalized with internal standards. Matrix data were exported as Excel files for the subsequent SVM analysis.

### Support vector machine

No processing procedure was performed for missing values. L2 normalization was used to normalize the data according to the following equation.

$$f_{i}=\frac{f_{i}}{\sqrt{\sum_{m=1}^{M}f_{m}^{2}}}$$

(where $f_{i}$ denotes the i-th dimension data for each sample, and M is dimension of the data.)

Given *N* samples ($x_{1},\;y_{1}$), ($x_{2},\;y_{2}$),($x_{N},\;y_{N}$), where $x_{i}\;$and $y_{i}$ were the data and label for i-th sample, the SVM found the “maximum-margin” or hyperplane that could divide those samples with the following equation.

$$\min_{w,b}\frac{{\left|\left|w\right|\right|}^{2}}{2},\;s.t.\left(w^{T}x_{i}+b\right)y_{i}\geq 1$$

As shown in above equation, the inferred w could be regarded as the importance weight for each feature. Based on w, we selected feature with higher importance. For the discriminating model constructed using the lipidomics data, we performed 2000 times of 4-fold cross-validation on the data from the discovery cohort and evaluated the classification performance. For the diagnostic model for MRM data, we constructed the SVM model using the training cohort, then tested and evaluated the classification performance using the test cohort and the validation cohort.

### Feature selection

To target selected features with information that is significant for classifying the MBGs versus NC, we computed the importance weight for each feature. Data in positive-ion (ESI+) and negative-ion (ESI-) modes were analyzed separately. Given all the normalized training data, denoted as **X∈R**^(107×1304(758))^, we employed SVM to infer the weight **W∈R**^(1×1304(758))^ for all features. Once the weight **W** was obtained**,** we treated the square value of ***W****_i_* as the importance weight of the i-th feature. All features were therefore sorted based on their weight value. Because a feature with a higher value has a greater influence, the validation operation was conducted to select Top-K importance features with the highest classification accuracy. In this work, Top-100 importance features were analyzed to generate predictive models for feature selection which was performed by selected the top-ranked feature one-by-one for evaluation, e.g., selecting the Top-N features among the N-th iteration. 500 times 4-fold cross-validations were carried out. Mean accuracies for each model (n = 100) in feature selection were calculated after 500 time of iterations.

### RNA-seq processing

Three pairs of glioblastoma samples and the corresponding adjacent normal tissues were taken during brain tumor resections and immediately frozen with liquid nitrogen. RNA was extracted using TRIzol. Sequencing libraries were then established using NEBNext UltraTM RNA Library Prep Kit for Illumina (NEB, USA), and the library preparations were sequenced on an Illumina HiseqX10 instrument (Illumina, Inc., San Diego, CA, USA) and 125 bp/150 bp paired-end reads were generated. For data analysis, we used GRCh38 (hg38) as the reference genome. Hisat2 v2.0.5 was used for genome mapping, and featureCounts v1.5.0 was used to count the read numbers of mapping. We then calculated the number of Fragments Per Kilobase of transcript sequence per Millions base pairs (FPKM) to estimate gene expression levels.

### Statistics

MATLAB R2018a and Prism Graphpad v 8.0 software were used for statistical analysis. For data from the targeted assay, t-SNE analysis was conducted with MATLAB using the *tsne* function. Principle component analysis (PCA) and hierarchy cluster analysis were performed using the MetaboAnalyst web service (https://www.metaboanalyst.ca/). Transcriptomic data were test for enrichment in curated KEGG pathways of glycerophospholipid metabolism (hsa00564) and glycerolipid metabolism (hsa00561) using GSEA software^28^ (1000 permutations).

### Ethics

This study was approved by the ethics committee at Beijing Tiantan Hospital (reference number: KY2014-021-02) as a part of the Neurosurgical Clinical Information and Biobanking Project (Brain Tumor Section) and conformed to the ethical guidelines of the 1975 Declaration of Helsinki. Written informed consent was obtained from each participant.

### Role of the funding source

The funding sources listed at the end of the manuscript did not have a role in the study design, sample collection, data analysis, result interpretation, or manuscript writing. The decision to submit the paper for publication was made only by the authors listed.

## Results

### Overview of the study scheme

**Figure 1:**
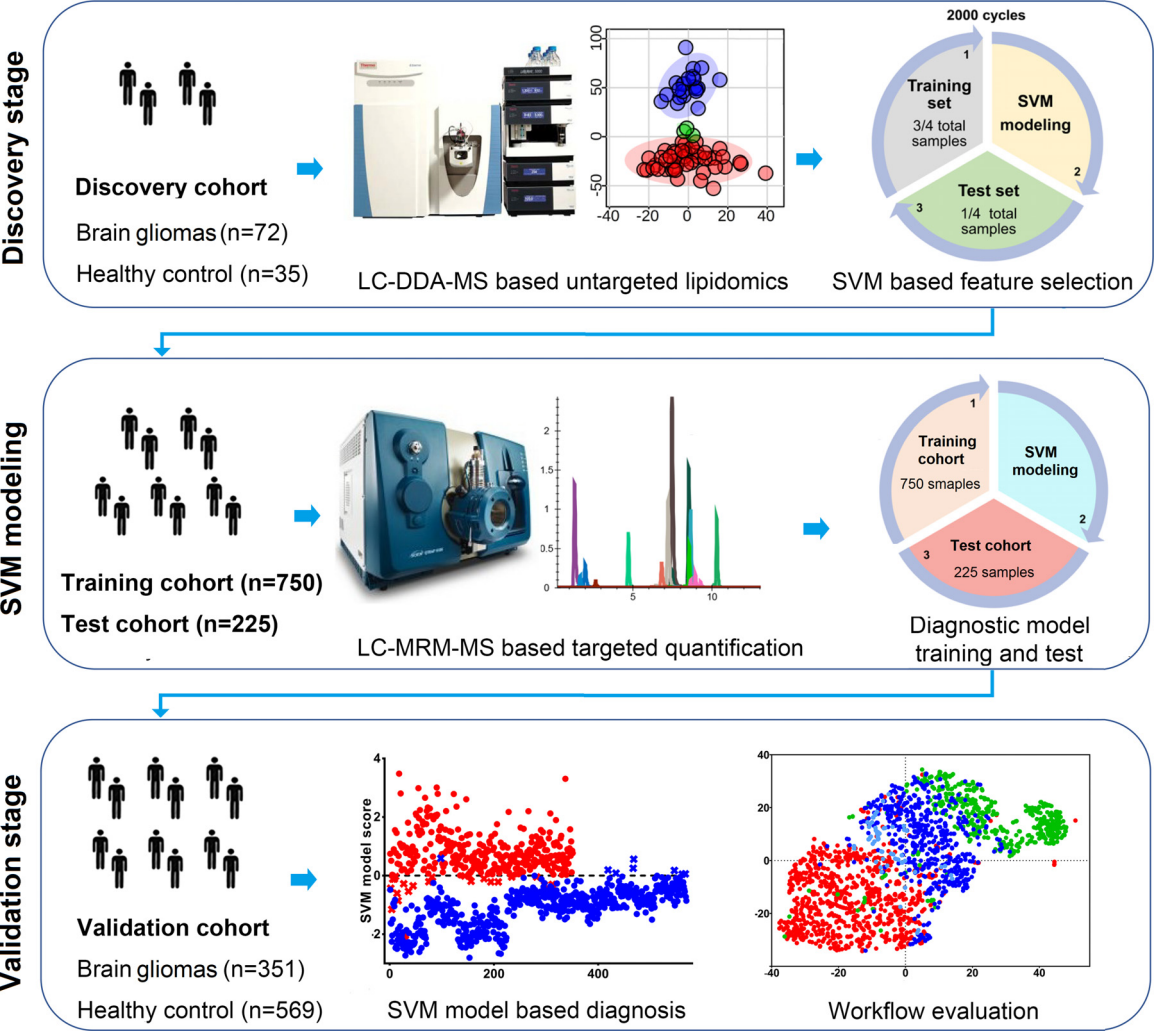
Overall workflow of the study. **Discovery stage:** LC-MS based untargeted lipidomics was initially used for plasma lipid profiling in a discovery cohort and results were processed by an SVM based discriminating model to screen for potential markers. The top ranked lipids were then selected as a panel of biomarkers for further validation. **SVM Modeling**: Independent training and test cohort were enrolled. LC-MRM-MS based targeted quantification assay method was developed and used for biomarker analysis. An SVM based diagnostic model was constructed using the training cohort and verified by the test cohort. **Validation stage**: The performance and clinical applicability of the diagnostic model was further evaluated by a newly collected validation cohort collected from multiple medical centers. The systematic bias and batch effects of the workflow were also evaluated.

In the present study we aimed to identify plasma biomarkers for diagnosis of MBGs. An overview of the study workflow is presented in Figure 1. LC-DDA-MS based untargeted lipidomics was initially used for plasma lipid profiling in a discovery cohort of 107 participants (72 MBGs, 35 NC). Lipidomic data were processed by an SVM-based feature selection procedure to screen for potential markers, and 11 top ranked lipids were ultimately selected as a panel of diagnostic biomarkers. Then an LC-MRM-MS-based targeted quantification method was constructed for this marker panel, and used for analysis of the training cohort (n=750, 385 MBGs and 365 NC) and test cohort (n=225, 115 MBGs and 110 NC). An SVM-based diagnostic model was built and tested with the results of the training cohort and the test cohort, respectively. Finally, we prospectively collected a validation cohort (n=920, 351 MBGs and 569 NC) at multiple medical centers to further evaluate the performance of the diagnostic model in an actual clinical setting.

### Lipidomic results and the discriminating model

**Figure 2:**
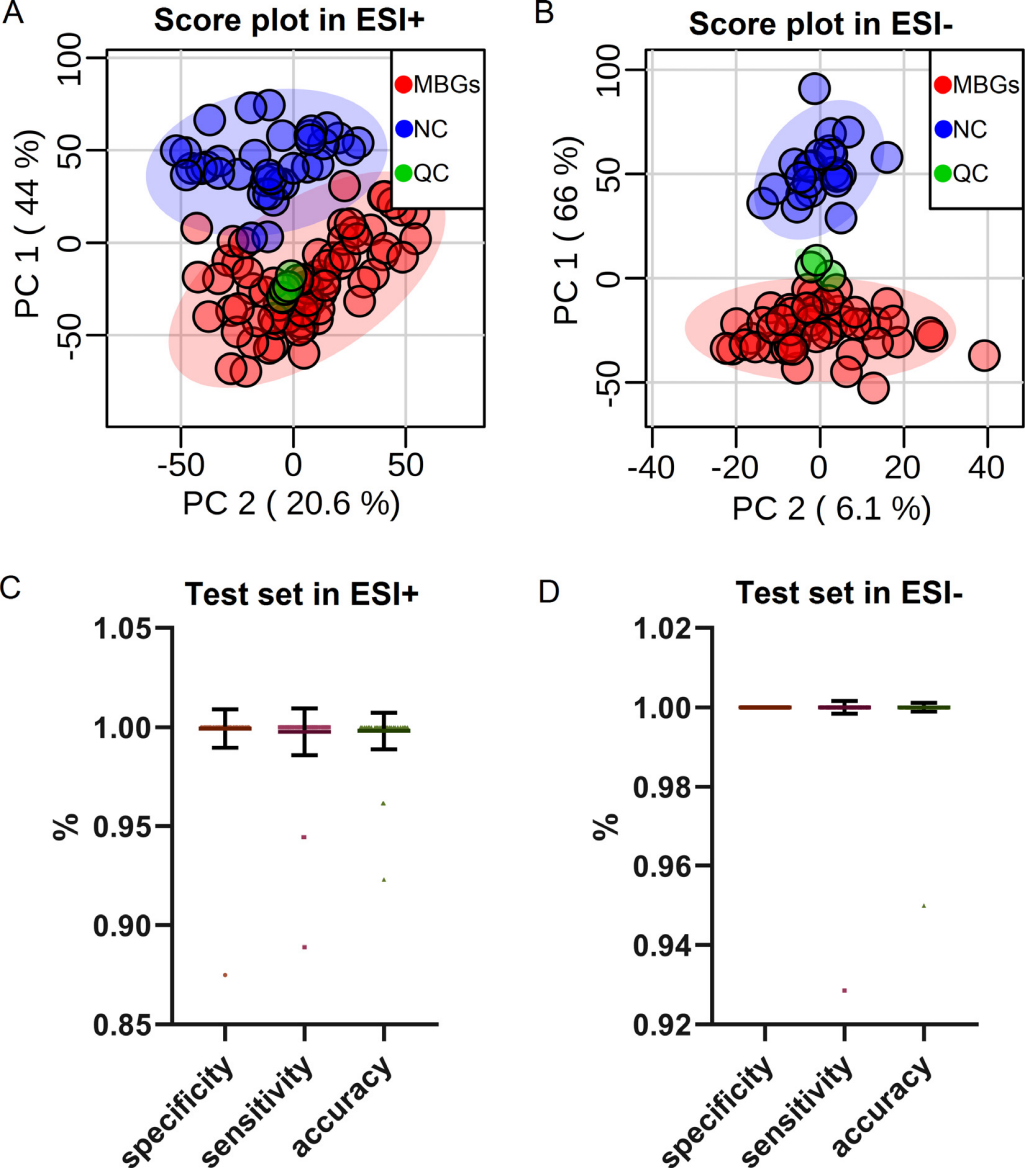
Overview of the results obtained for the biomarker of discovery stage. (**A-B**) PCA score plots of the untargeted lipidomic result in A) positive ion mode (ESI+) and B) negative ion mode (ESI-). Sample groups are represented by colors: malignant brain gliomas (MBGs, n=72), red; healthy control (NC, n=35), blue; quality control (QC), green. (**C-D**) Summary of model parameters in terms of specificity, sensitivity and accuracy for the test set in C) positive ion mode (ESI+) and D) negative ion mode (ESI-). N=2000 iterations for SVM model construction; each dot represents data for one iteration of specificity/sensitivity/accuracy in SVM evaluation, and data are presented as means ± SD.

For the discovery cohort, a total of 1304 plasma lipids belonging to 16 lipid species, and 758 plasma lipids belonging to 27 lipid species were identified at MS2 level in ESI+ and ESI- ion modes, respectively (Figure S1). First, PCA score plots were used for data quality evaluation in which QC samples clustered well in both ESI+ (Figure 2A) and ESI- (Figure 2B) ion modes. Samples in MBGs and NC groups showed trends of separation. To further discriminate MBGs from NC and reveal the most powerful lipid markers, we employed an SVM-based discriminative model and feature selection procedure with all detected lipids. Three quarters of the total samples were randomly chosen as a training test set for modeling while the remaining 1/4 of the samples were used as the test set, and the procedure was repeated 2000 times to obtain an average result. After 2000 iterations, the overall presentation of model parameters of the test set in ESI+ (Figure 2C) and ESI- (Figure 2D) modes are shown in terms of specificity, sensitivity and accuracy. The mean accuracy was 0.999 (95% confidence interval (CI), 0.997 to 1.000) with a sensitivity of 0.998 (95% CI, 0.997 to 0.999) and a specificity of 0.999 (95% CI, 0.998 to 1.000) in the data from ESI+ mode (Figure 2C and Table 2). The mean accuracy was 1.00 (95% CI, 1.00 to 1.00) with a sensitivity of 1.00 (95% CI, 1.00 to 1.00) and a specificity of 1.00 (95% CI, 1.00 to 1.000) in the data from ESI- mode (Figure 2D and Table 2). Because the plasma samples in the MBGs group were collected randomly from either arterial or venous blood during surgery under anesthetic, we also compared the lipid profiles between paired arterial blood (collected during surgery under anesthetic) and venous blood (collected before surgery without anesthetic) samples collected from 31 MBGs patients, to evaluate any systematic biases caused by disease irrelevant factors. No significant changes were found between arterial and venous derived plasma, or between different states of anesthesia (Figures. S2 and S3).

**Table 2:** Parameters of the SVM models for DDA and MRM datasets.

		DDA Dataset				
	Training set			Test set		
ESI+	specificity	sensitivity	accuracy	specificity	sensitivity	accuracy
mean	1.000	1.000	1.000	0.999	0.998	0.999
95% CI down	1.000	1.000	1.000	0.998	0.997	0.997
95% CI up	1.000	1.000	1.000	1.000	0.999	1.000

ESI-	specificity	sensitivity	accuracy	specificity	sensitivity	accuracy

mean	1.000	1.000	1.000	1.000	1.000	1.000
95% CI down	1.000	1.000	1.000	1.000	1.000	1.000
95% CI up	1.000	1.000	1.000	1.000	1.000	1.000

	MRM Dataset					

	Training cohort	Test cohort	Validation cohort			

Accuracy	0.9467	0.9244	0.9641			
Specificity	0.9534	0.9727	0.9859			
Sensitivity	0.9403	0.8783	0.9288			
AUC of ROC	0.9877	0.9869	0.9866			

### Feature selection and MRM method development

**Figure 3:**
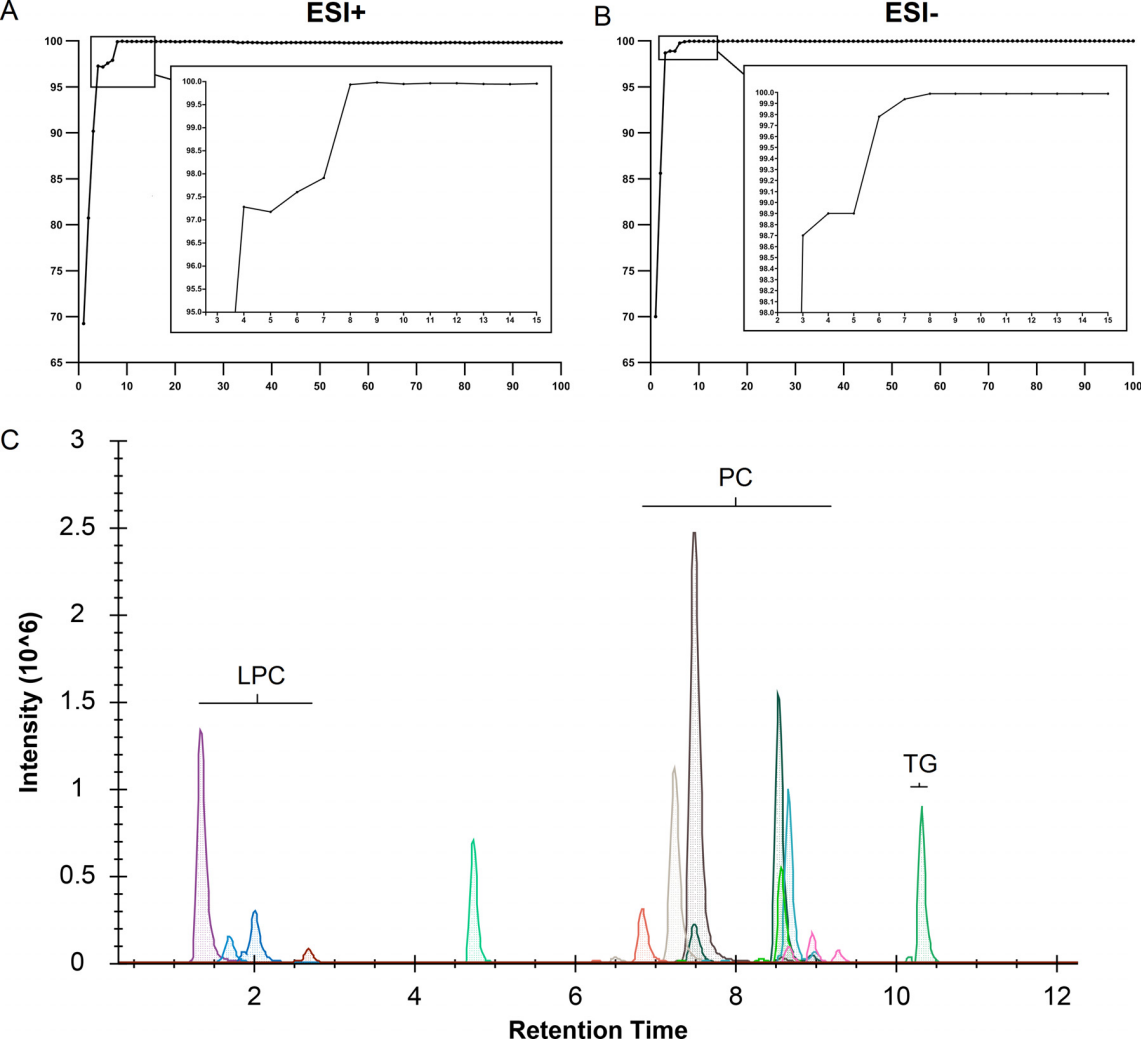
Feature selection and MRM method construction for candidate biomarkers. (**A-B**) Mean accuracies on the test set for models in A) positive ion mode (ESI+) and B) negative ion mode (ESI-) after 500*100 times iterations (500 iterations for the top-100 feature set). The x-axis represents feature numbers and y-axis represents discriminating accuracy. (**C**) XICs of the eleven targeted lipids analyzed by the 19 min. LC-MRM-MS assay method. The x-axis represents the retention time of the liquid chromatography and the y-axis represents the ion intensity detected by the mass spectrometer.

After the construction of the SVM discriminating model, we performed feature selection to retrieve candidate biomarkers. After 100*500 times of iterations, mean accuracies on the test set for each model are shown in Figure 3A (ESI+ mode) and Figure 3B (ESI- mode). With 8 features, the models achieved accuracies of more than 99%; consequently, we chose the top 8 features from ESI+ and ESI- modes separately as candidate features whose identities were further verified against commercially available chemical standards (Figs. S4 and S5). As can be seen from the detailed information summarized in Table 3, most of the top ranked features in ESI+ and ESI- modes overlapped, demonstrating the reliability of the feature selection procedure. Collectively, 11 non-redundant lipids (LPC 16:0, LPC 18:0, LPC 18:2, PC 16:0-18:1, PC 16:0-18:2, PC 16:0-20:4, PC 16:0-22:6, PC 18:0-18:1, PC 18:0-18:2, PC 18:0-22:4 and TG 18:1-18:2-18:3) were chosen as candidate markers after combining the top ranked features from both ESI+ and ESI- modes. Then, an LC-MRM-MS based assay method was developed in which the panel of 11 lipids could be analyzed in a 19 min LC-MS run in an ESI+ ion mode, as the XICs show in Figure 3C.

**Table 3:** Information of the 11 lipids markers for malignant brain gliomas.

Lipid name	Detected in DDA ESI+	Detected in DDA ESI-	Matched with MS/MS in database	Validated by chemical standard	Rank in SVM model in ESI+	Rank in SVM model in ESI-
LPC 16:0	✔	✔	✔	✔	1	2
LPC 18:0	✔	✔	✔	✔	2	1
LPC 18:2	✔	✘	✔	✘	6	-
PC 34:1; PC 16:0-18:1	✔	✔	✔	✔	4	4
PC 34:2; PC 16:0-18:2	✔	✔	✔	✔	15	6
PC 36:4; PC 16:0-20:4	✔	✔	✔	✔	14	7
PC 38:6; PC 16:0-22:6	✔	✔	✔	✔	5	11
PC 36:1; PC 18:0-18:1	✘	✔	✔	✔	-	5
PC 36:2; PC 18:0-18:2	✔	✘	✔	✔	3	-
PC 38:4; PC 18:0-20:4	✔	✔	✔	✔	7	8
TG 18:1-18:2-18:3	✔	✘	✔	✘	8	-

### Diagnostic model training and test

**Figure 4:**
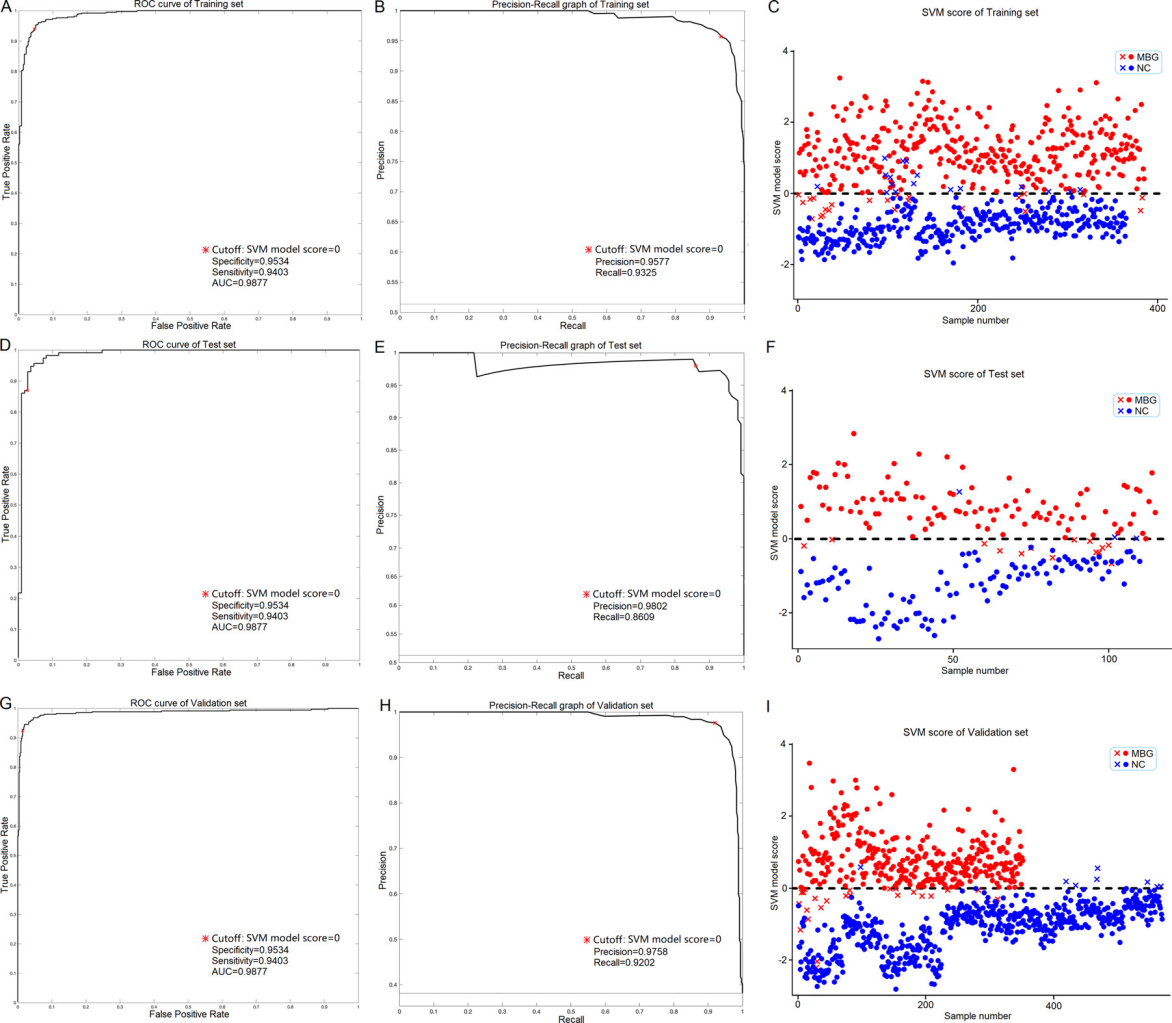
Overview of the results of validation stage. (**A, D, G**) ROC curves of the training cohort (**A**, n=750), test cohort (**D**, n=225) and validation cohort (**G**, n=920) processed by the SVM based diagnostic model. The asterisk sign denotes the cutoff (SVM model score = 0). (**B, E, H**) Precision-Recall curves of the training cohort (**B**), test cohort (**E**) and validation cohort (**H**) processed by the SVM based diagnostic model. The asterisk sign denotes the cutoff (SVM model score = 0). (**C, F, I**) Score distribution of the samples in the training cohort (**C**), test cohort (**F**) and validation cohort (**I**). The score of each sample was output by the SVM based diagnostic model. Sample groups were presented by colors (red for MBGs and blue for NC). Samples with a score greater than 0 were predicted as cancer patients. Correctly grouped samples were presented as circle and incorrectly grouped samples were presented as cross.

To better adapt the application of this lipid marker panel to a clinical setting, we first constructed an SVM based diagnostic model using the MRM assay result of the training cohort. As shown in Table 2 and Figure 4A and B, the model achieved a high discriminating performance with an accuracy of 0.9467, a specificity of 0.9534, a sensitivity of 0.9430 and an AUC of 0.9877. The model scoring result of the training cohort is shown in Figure 4C in which a score greater than 0 represents a prediction of cancer. Furthermore, the reliability of the diagnostic model was tested and verified using the test cohort, and an accuracy of 0.9244, a specificity of 0.9727, a sensitivity of 0.8783 and an AUC of 0.9869 were achieved (Table 2 and Figure 4D-F). The discriminating performance of the diagnostic model in the training and test cohorts demonstrates the reliability of our lipidomic workflow and feature selection procedure, and also establishes the applicability of this lipid marker panel in MBGs diagnosis.

### Diagnostic model application and evaluation

**Figure 5:**
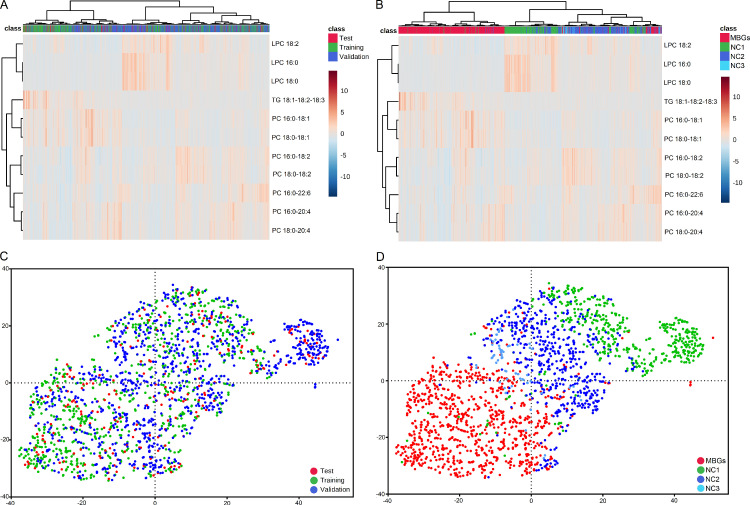
Overview of the batch effects and systematic bias of the assay method. (**A** and **C**) The heat map of hierarchical clustering analysis (**A**) and scatter plot of t-SNE analysis (**C**) of the MRM datasets in terms of cohorts. Different cohorts were marked by colors (green, training cohort; red, test cohort; blue, validation cohort). (**B** and **D**) The heat map of hierarchical clustering analysis (**B**) and scatter plot of t-SNE analysis (**D**) of the MRM datasets in terms of medical centers. Samples collected at different medical centers were marked by colors (red, malignant brain gliomas samples from Tiantan hospital (MBGs); green, healthy control samples from Haidian hospital (NC1); blue, healthy control samples from Peking University Third Hospital (NC2); light blue, healthy control samples from Tiantan Hospital (NC3)).

To further evaluate the diagnostic performance of the marker panel in an actual clinical setting, we tested it on a freshly collected validation cohort containing 920 participants from three medical centers. As shown in Table 2 and Figure 4G and H, the model continued to achieve a good discriminating performance with this new cohort with an accuracy of 0.9467, a specificity of 0.9534, a sensitivity of 0.9430 and an AUC of 0.9877. The model scoring result of the validation cohort is shown in Figure 4I in which most samples were assigned to the correct group. Possible batch effects and systematic bias of the data were also evaluated. As can be seen in the heat map (Figure 5A) and t-SNE scatter plot (Figure 5C), no differences in distribution were observed with respect to the training, test and validation cohorts, demonstrating the reproducibility of the analytical workflow. In terms of medical centers, even though differences in distribution can be observed between samples derived from different medical centers (Figure 5B and D), the MBGs group could still be readily separated from the NC group with a high degree of accuracy.

### RNA-sequencing analysis of MBGs tissue

To investigate the potential mechanism(s) linking the plasma lipid markers to MBGs, we profiled the RNA expression levels in samples from MBGs patients as well as in normal brain tissues, and analyzed the gene expression levels in two relevant metabolic pathways (glycerophospholipid metabolism for LPCs and PCs, and glycerolipid metabolism for TG) using the GSEA algorithm. As shown in Table S2 and Figure S6, these two pathways were significantly dysregulated in MBGs tissues (adjusted P-value < 0.01).

## Discussion

In this study, we combined LC-MS based lipidomic analysis with an SVM-based ML algorithm to screen for plasma lipid biomarkers for MBGs diagnosis. A panel of 11 lipids was ultimately identified as biomarkers and a diagnostic model was built and evaluated with independent patient cohorts. Our findings provide a powerful and non-invasive diagnostic method for malignant brain gliomas, and at the same time they provide a study workflow that combines lipidomics and ML that can be used to efficiently and reliably screen for biomarkers of diseases.

Because of its extensive metabolite coverage and high degree of sensitivity, untargeted metabolomics and lipidomics have become powerful strategies for plasma metabolite/lipid profiling, and have been widely used in biological studies and biomarker screening research.^29^^,^^30^ In the discovery stage of this study, the reliability of our lipidomic results was firstly evaluated by PCA score plots and verified by well clustered QC samples in both ESI+ and ESI- modes. Even though obvious trends of separation between MBGs and NC groups were observed by PCA, the high complexity of the biomolecular content of plasma made it challenging to interpret the results as well as retrieving the most significant biomarkers from thousands of candidates. Thus, we further employed an SVM-based discriminating model to screen the lipidomic results. We chose SVM model concerning the following reasons:1) SVM is a robust classifier for two-class classification, especially for the training dataset consisting of a small sample size and dimension,^31^^,^^32^ thus it is suitable to our untargeted lipidomic data. 2) the weight of feature generated by SVM model can achieve feature selection, which is practical for selecting biomarker panels for following targeted analysis. Compared with the conventional ways for biomarker searching in metabolomics (such as statistical *P*-value, fold change between groups and VIP values in PLS-DA/OPLS-DA models), the SVM-based ML algorithm we used can not only rank all the features according to their relative contribution for discriminating different groups, but can also provide overall accuracy in terms of numbers of selected features, thus making it practical to achieve a high accuracy with the least number of biomarkers. What is more, as reported by Mahadevan and colleagues, SVM is able to give a better predictive model with a fewer number of features when compared to PLS-DA.^21^ Among the 11 chosen lipid markers, many were detected and top-ranked in the model of both ESI+ and ESI- modes, which also demonstrates the reliability of our lipidomic workflow as well as the SVM-based feature selection procedure.

To validate the panel of markers and ultimately apply it in a clinical setting, we aimed to develop a high throughput assay which could accurately quantify the 11 lipid markers in a large sample cohort. Thus, an LC-MRM-MS based assay was developed with optimized analytical parameters with the assistance of commercial chemical standards. All 11 lipid targets were uniformly analyzed in ESI+ ion mode to take advantage of the higher signal response for LPCs and PCs. Finally, this optimized method reliable quantified the markers in 19 min. This MRM-based high throughput assay method could analyze one sample in about two hours including sample preparation and data processing time, and batch processing of samples could further shorten the average analyzing time for each sample to less than 30 min. This advantage makes it applicable and practical to be used in clinically related diagnostic applications involving a large number of samples. With the MRM assay result of the training cohort, we established the SVM based diagnostic model and tested it with a test cohort. To better evaluate the model performance in an actual clinical setting, we enrolled a newly collected validation cohort comprised of participants from multiple medical centers, and results from this analysis provide further validation of the robustness of this diagnostic model. We also evaluated the systematic biases and batch effects relevant to our study workflow. Systematic bias commonly exists in sample conditions, we compared patient plasma samples of different origin (artery or vein) and anesthesia status, but no differences were observed. Batch effects are often introduced during analytical procedures such as lipid extraction and LC-MS analysis, so we included internal standards for normalization, so that possible batch effects could be excluded. As shown in Figure 5, no batch effects existed between the training, test and validation cohorts. Interestingly, we observed some apparent bias between samples from different medical centers, which may result from difference in sample collection procedures or storage conditions. This bias could not be completely eliminated in actual practice, but our diagnostic method has successfully discriminated MBGs groups from NC groups from three different medical centers with high accuracy in spite of the existing bias, thus further demonstrating its applicability. In this study we excluded patients and healthy controls who had systematic diseases such as diabetes, cardiovascular diseases or metabolic disorders. But patients only with high LDL or TG etc. were not excluded because these situations are common in population and clinical practice. To better exclude the influence of these brain glioma-unrelated factors, we managed to include large sample cohort, and to collect samples from different medical centers in the validation stage. Our result showed good diagnostic power in the validation cohort, proving the common brain glioma-unrelated factors could not influence the performance of the diagnostic model significantly.

An increasing number of studies have employed ML in medically related applications to take advantage of its high sensitivity and powerful diagnostic potential.^33^^,^^34^ For example, deep neural networks have been used to process Raman histology figures for near real-time intraoperative brain tumor diagnosis.^35^ With respect to non-invasive diagnostic tests for tumors, AI has also shown several major advantages, such as those described by Jacob and colleagues who employed ML in a search for a blood DNA marker for early stage lung cancer detection,^24^ Gregory and colleagues also applied AI in their search for microbe-derived DNA fragments in blood for the detection of several kinds of cancer,^25^ as did Nickolas and colleagues who combined ctDNA and protein markers to better detect cancer.^1^^,^^5^ However, all of these studies have focused on the analysis of cell-free DNA or proteins in the blood, and thus far, no studies have focused on interpreting the linkage between the blood lipidome and cancer onset using AI techniques, such as we have performed in the present study. Here, we implemented a SVM based ML algorithm in two steps: the first was to establish the discriminating model for efficient and reliable candidate marker selection from the untargeted lipidomic data (from the discovery cohort); the second step was to establish a diagnostic model to process MRM data (from the training, test and validation cohorts) for feasible and reliable diagnosis of MBGs. Our selected lipid marker panel and constructed diagnostic mode showed excellent discriminating power for MBGs, and applicability and potential for the strategy of combining lipidomics and AI techniques in biomarker development. The developed markers and assay method may have more potential applications such as evaluating reduction of tumor load, monitoring progression or recurrence or predicting prognosis for MBGs patients. But these potential applications need to be further validated by corresponding cohorts before conclusions can be made.

The candidate lipid markers we found belonged to three lipid species (LPC, PC and TG). In cells, LPC, PC and TG are involved in pathways of glycerophospholipid metabolism and glycerolipid metabolism, and our RNA sequencing results showed that these two pathways were perturbed in plasma from MBGs patients, suggesting that changes in the plasma levels of these lipids may reflect a perturbed lipid metabolism in MBGs tissue. Compared with normal cells, tumor cells usually consume more saturated and monounsaturated LPCs which are carriers of fatty acids.^36^ Decreased plasma LPC levels has been found in various cancers, and shifting LPC from plasma to tumor tissue has been considered as a way to support sustained tumor proliferation.^37^, ^38^, ^39^, ^40^ Aberrant PC metabolism and up-regulated PC levels have also been found in several kinds of cancer cells, such as epithelial ovarian cancer,^41^ colorectal cancer^42^ and lung cancer.^43^ Lyso-PC acyltransferased (LPCAT) family members have been reported to be increased to catalyze the processes from LPCs to PCs in cancer cells.^42^ Similarly, our RNA sequencing analyses showed up-regulated levels of LPCAT1 and LPCAT3 in MBGs tissue. TG has also been reported to accumulate in more aggressive lung cancers^44^ as well as during the epithelial-to-mesenchymal transition process of prostate cancer cells.^45^ Over all, changes in TGs and phospholipids such as PCs and LPCs may result in changes in cell membrane composition or cellular metabolic status. This in turn could influence cell proliferation, viability or development of tumors. The plasma lipid markers proposed by our current study are dysregulated but the molecular mechanism(s) responsible for these changes could not be fully explained just by the RNA sequencing results because these represent just a “snapshot” of the metabolic status in MBGs cells. Additional researches such as transcriptomic, proteomic and metabolomic analyses of tumor tissues in larger sample size are needed to uncover the mechanistic basis for these changes.

The approach described here has several important limitations. First, given that samples collected from different hospitals may vary in a manner by which they were collected and/or stored, the validation cohort from multiple centers would be better choice for biomarker performance evaluation. Because of the practical challenges of enrolling MBGs patients in this study, all 851 patients in MBGs groups came from the same medical center (Tiantan Hospital). However, NC enrolled in this study came from three medical centers including Tiantan Hospital itself, and the result showed that even though a partial bias exists in samples collected from different medical centers, our panel of proposed lipid biomarkers could still perform well enough to discriminate MBGs patients from NC. The second limitation of our study relates to the MRM assay method developed for biomarker analysis of the training, test and validation cohorts. An assay method able to absolutely quantify the 11 lipids would be the ideal choice because it can provide concentrations of the plasma biomarkers. However, no ideal blank matrix (a sample which consists of all plasma components except only the 11 target lipids on which we focused) is commercially available. Even though a kind of simulated blank plasma or charcoal-treated plasma samples have been used for absolute quantification of endogenous molecules in biological samples,^46^^,^^47^ it remains a compromise rather than an ideal solution. Consequently, in the present study, the MRM assay workflow we used only employed internal standards to normalize the technique errors and batch effects in sample preparations, and no standard curves were employed in the quantification. Thus, this assay method can be viewed as a “semi-absolute” quantitative method. At this stage, we believe that our assay method could fulfill current needs (biomarker validation and performance evaluation in large cohorts) in terms of analytical reliability, stability and repeatability between batches.

## Conclusions

The present study integrated SVM based ML algorithm into conventional lipidomic biomarker screening workflow, revealing a panel of 11 potential lipid markers for MBGs. These lipid markers and related algorithm have shown high accuracy in identifying MBGs blood samples, demonstrating their high value in disease diagnosis. Advantage of this diagnostic strategy includes non-invasive sample collection, quick analysis and high accuracy. What is more, our study shows the applicability, advantages and broad prospects of a strategy using ML algorithms and metabolomics in disease related biomarker screening.

## Contributors

Y.Y., L.Z., N.J. and J.Z. conceived the study and designed major experiments. H.S. and Y.Yuan. made sample preparation. N.J. performed sample collection and clinical diagnosis. J.Z. performed the lipidomic profiling experiments, analyzed the data and established the targeted lipid profiling. G.W. performed machine learning and analyzed the data. C.Y. performed the RNA sequencing experiments and analyzed the data. Y.Z. Y.J. and Z.Z. helped interpret the results. The manuscript was written by J.Z., N.J. L.Z. and Y.Y.

## Declaration of interests

The authors declare that they have no competing interests.

## Data sharing statement

Data are available from the authors upon reasonable request. Raw mass spectrometry data was deposited in MassIVE database with the ID: MSV000085693. RNA-Seq data was deposited in NCBI SRA with the ID: PRJNA649346. SVM code and example data was supplied in the supplementary materials.

## Appendix Supplementary materials

Image, application 1

Image, application 2

Image, application 3
