# Supplementary material for: Metabolic detection of malignant brain gliomas through plasma lipidomic analysis and support vector machine-based machine learning
Source: eBioMedicine. 2022 Jun 7;81:104097. doi: 10.1016/j.ebiom.2022.104097 (PMC9189781; doi:10.1016/j.ebiom.2022.104097)
Supplement: Supplementary file 1 [file mmc1.docx]

**List of supplementary files:**

1. Supplementary materials.docx
2. SVM code and data.rar
