# Supplementary material for: Metabolic detection of malignant brain gliomas through plasma lipidomic analysis and support vector machine-based machine learning
Source: eBioMedicine. 2022 Jun 7;81:104097. doi: 10.1016/j.ebiom.2022.104097 (PMC9189781; doi:10.1016/j.ebiom.2022.104097)
Supplement: Supplementary file 2 [file mmc2.docx]

**Supplementary materials for:**

**Metabolic detection of malignant brain gliomas through plasma lipidomic analysis and support vector machine-based machine learning**

Juntuo Zhou^1,2,4^, Nan Ji^3,4^, Guangxi Wang^2^, Yang Zhang^3^, Huajie Song^2^, Yuyao Yuan^2^, Chunyuan Yang^2^, Yan Jin^1^, Zhe Zhang^3^, Liwei Zhang^3*^ & Yuxin Yin^1,2*^

^1^Institute of Precision Medicine, Peking University Shenzhen Hospital, Shenzhen 518036, China;

^2^Institute of Systems Biomedicine, Department of Pathology, School of Basic Medical Sciences, Peking-Tsinghua Center for Life Sciences, Peking University Health Science Center, Beijing 100191, China;

^3^Department of Neurosurgery, Beijing Tiantan Hospital, Capital Medical University, National Clinical Research Center for Neurological Diseases, Beijing 100070, China;

^4^These authors contributed equally to this work.

**Supplementary Materials**

Figure S1. Lipid species identified in the discovery stage.

Figure S2. Comparison between plasma lipidomic profiles deriving from artery and vein blood.

Figure S3. The expressive levels of the eleven lipid markers in plasma samples derived from artery and vein blood.

Figure S4a. Identity verification of the lipid markers by chemical standards.

Figure S4b. Identity verification of the lipid markers by chemical standards.

Figure S5a. Identity verification of the lipid markers by chemical standards.

Figure S5b. Identity verification of the lipid markers by chemical standards.

Figure S6a. The enrichment plot of GSEA.

Figure S6b. The heat map presenting the expressive profiles of genes in the pathway of glycerophospholipid metabolism and glycerolipid metabolism.

Table S1. Parameters of the MRM transitions.

Table S2. GSEA result of the RNA sequencing analysis in terms of two marker related KEGG pathways.

Table S3a. Expressive profiles of genes in glycerophospholipid metabolism.

Table S3b. Expressive profiles of genes in glycerolipid metabolism.

pid metabolism.

**Figure S1. Lipid species identified in the discovery stage.**

**
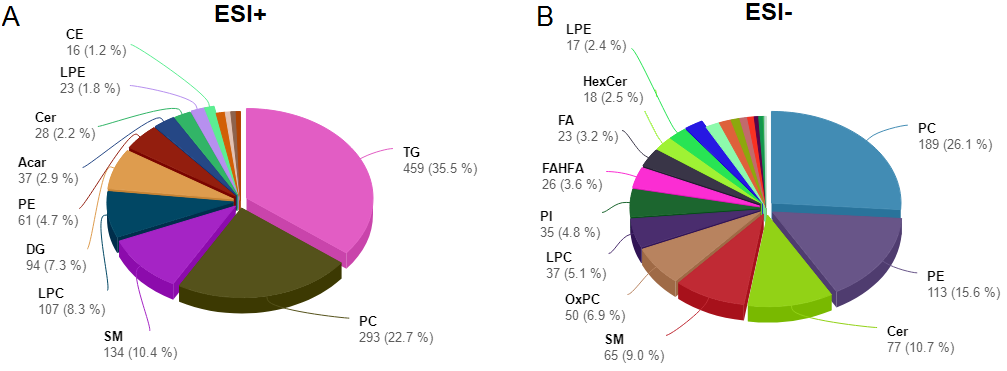
**

**Figure S1.** **Lipid species identified in the discovery stage.**

Lipid species identified in the discovery stage in ESI+ (**A**) and ESI- (**B**) modes by untargeted lipidomics. Abbreviations: CE, Cholesteryl ester; Cer, Ceramide; HexCer, hexosylceramides; FFA, Free fatty acid; FAHFA, branched fatty acid esters of hydroxy fatty acids; OxFA, Oxidized fatty acid; LPC, Lysophosphatidylcholine; LPE, Lysophosphatidylethanolamine; PC, Phosphatidylcholine; p-PC, Ether-linked phosphatidylcholine; OxPC, Oxidized phosphatidylcholine; ACar, Acylcarnitine; PE, Phosphatidylethanolamine; p-PE, Ether-linked phosphatidylethanolamine; DG, Diacylglycerol; TG, Triacylglycerol; SM, Sphingomyelin; PI, Phosphatidylinositol.

**Figure S2. Comparison between plasma lipidomic profiles deriving from artery and vein blood.**

**
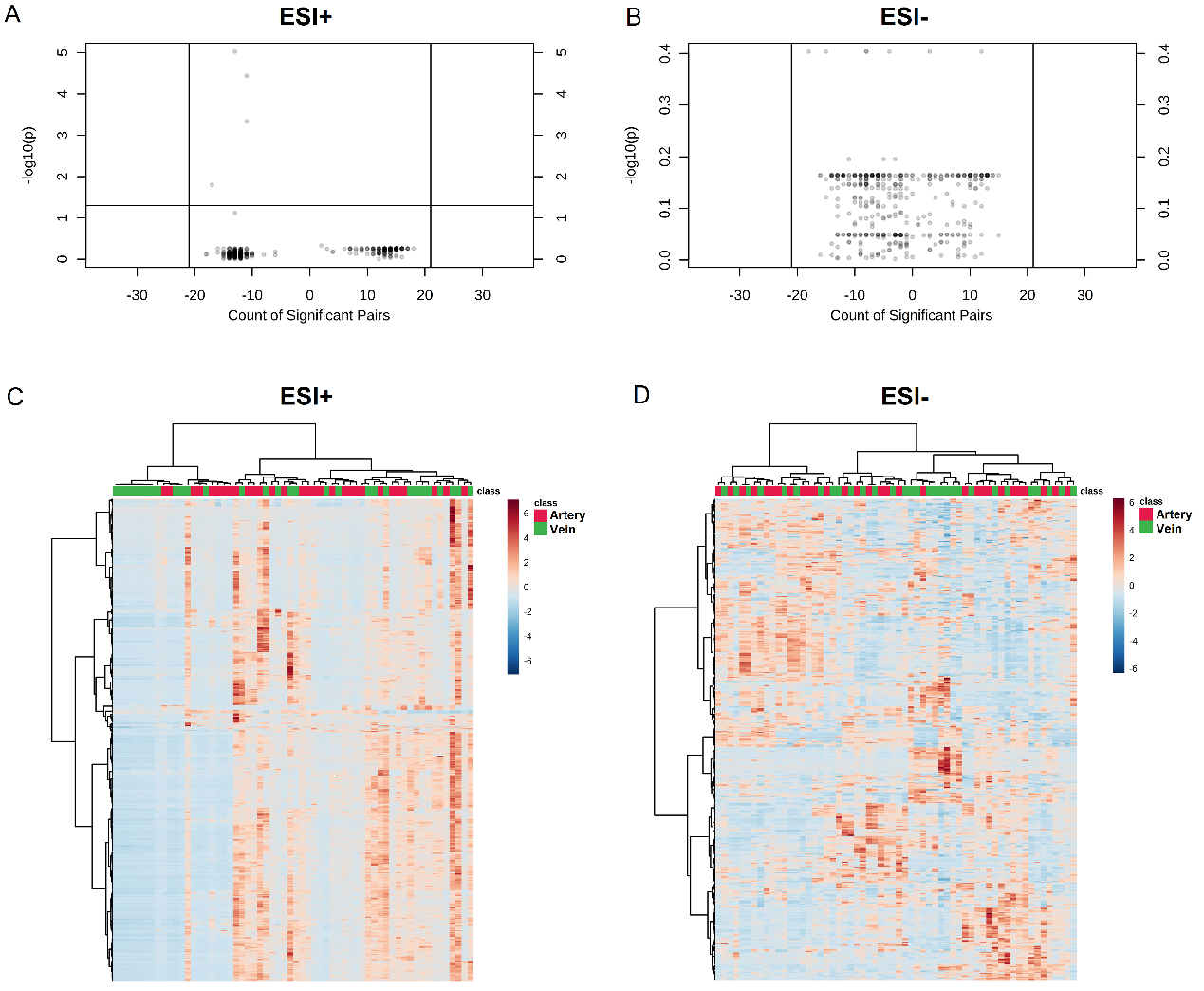
**

**Figure S2. Comparison between plasma lipidomic profiles deriving from artery and vein blood.**

(**A-B**) Scatter plots representing the result of paired t-test using data from ESI+ (**A**) and ESI- (**B**). *P* value of 0.05 and count of significant pairs of 70% were set as cutoffs, and no difference was found. (**C-D**) Scatter plots representing the lipid expression profiles using data from ESI+ (**C**) and ESI- (**D**). No difference was found.

**Figure S3. The expressive levels of the eleven lipid markers in plasma samples derived from artery and vein blood.**


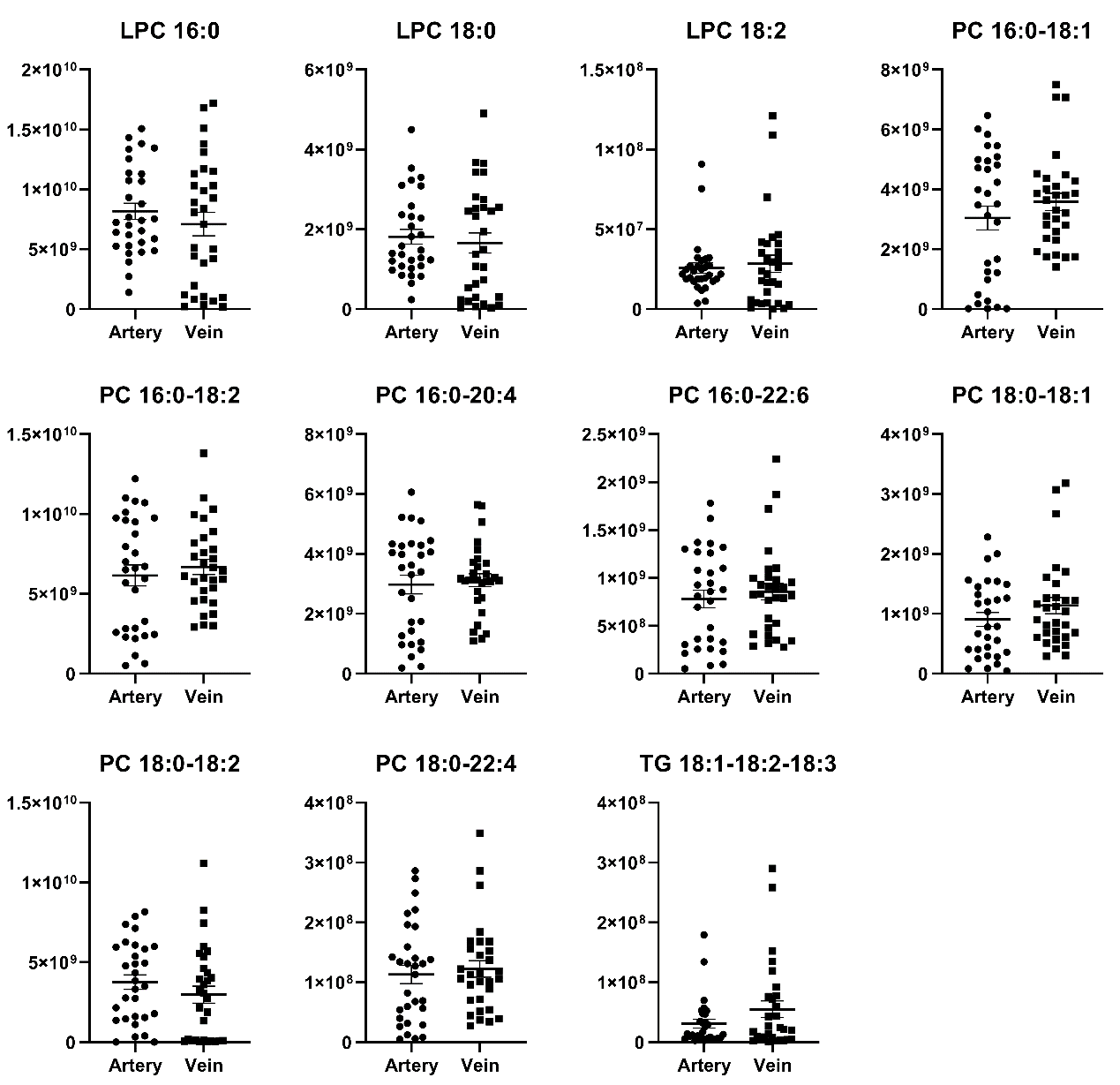


**Figure S3. The expressive levels of the eleven lipid markers in plasma samples derived from artery and vein blood.**

Data are presented as means ± SD. No difference was found.

**Figure S4a. Identity verification of the lipid markers by chemical standards.**

**
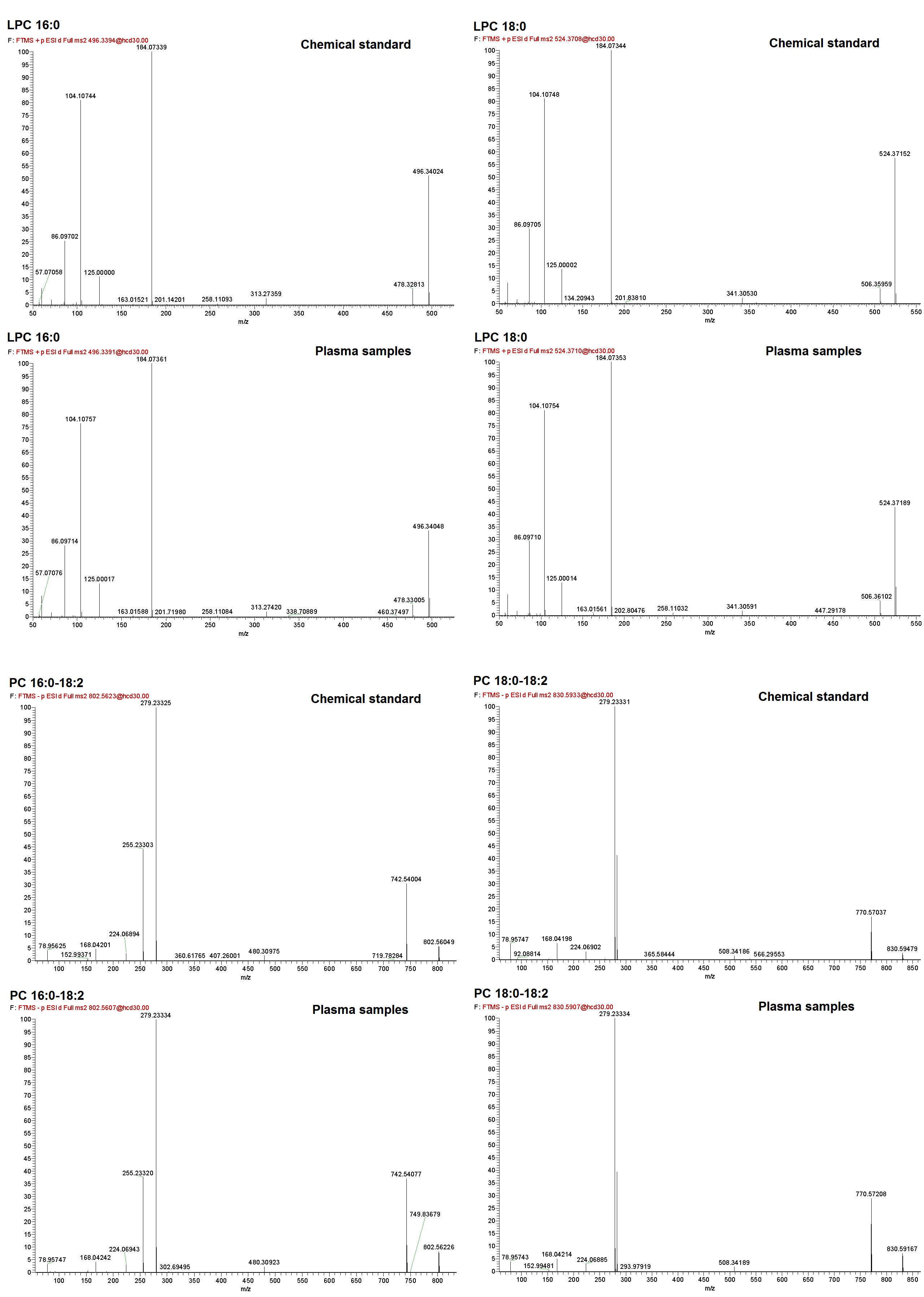
**

**Figure S4a. Identity verification of the lipid markers by chemical standards.** Comparison of MS2 spectra of LPC 16:0, LPC 18:0, PC 16:0-18:2 and PC 18:0-18:2 between chemical standard and the counterpart in plasma (lipidomics result). The MS2 spectra were acquired in DDA mode on a Q-Exactive LC-MS platform.

**Figure S4b. Identity verification of the lipid markers by chemical standards.**

**
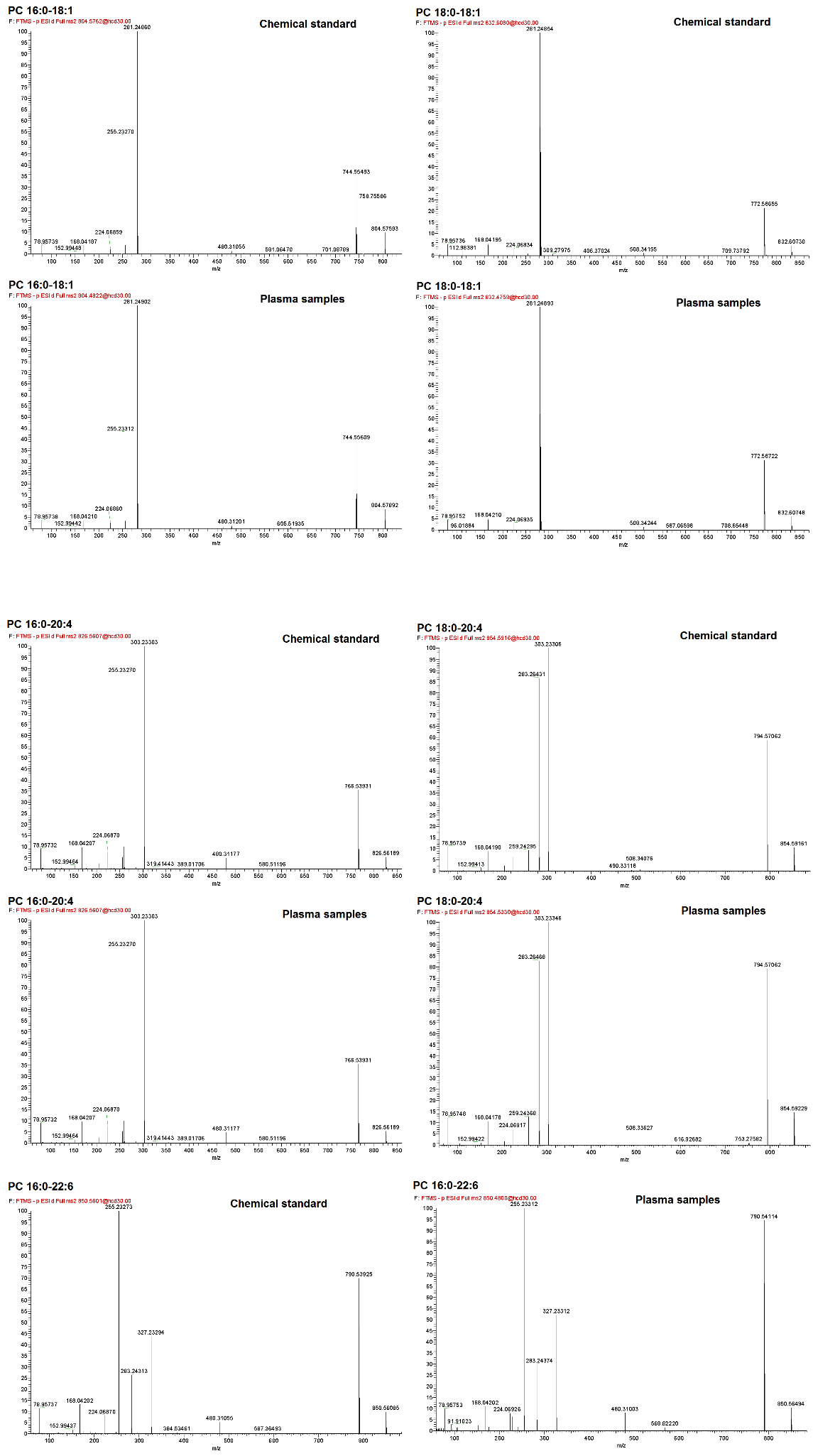
**

**Figure S4b. Identity verification of the lipid markers by chemical standards.** Comparison of MS2 spectra of PC 16:0-18:1, PC 18:0-18:1, PC 16:0-20:4 and PC 18:0-20:4 and PC 16:0-22:6 between chemical standard and the counterpart in plasma (lipidomics result). The MS2 spectra were acquired in DDA mode on a Q-Exactive LC-MS platform.

**Figure S5a. Identity verification of the lipid markers by chemical standards.**

**
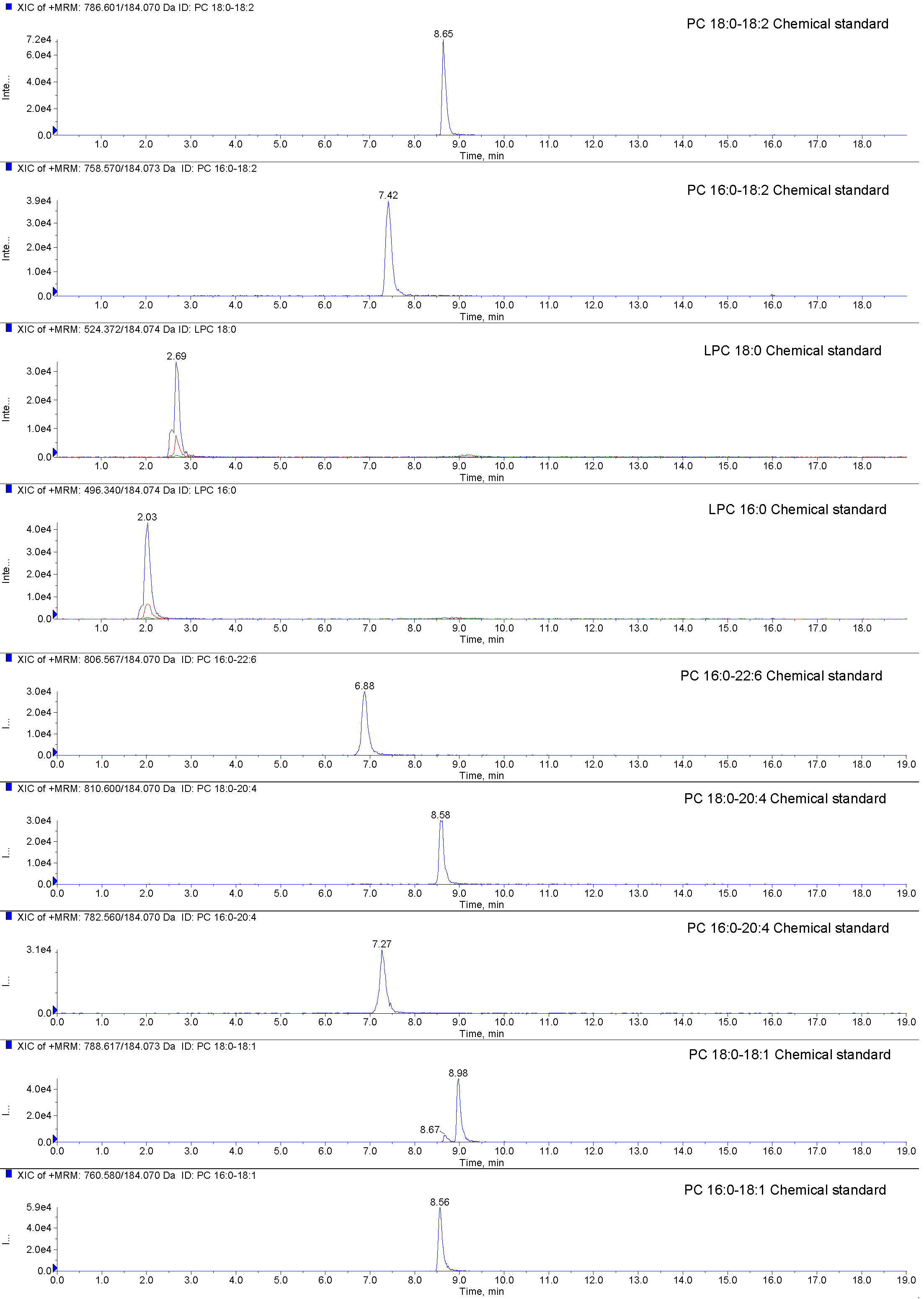
**

**Figure S5a. Identity verification of the lipid markers by chemical standards.**

XIC and retention time of LPC 16:0, LPC 18:0, PC 16:0-18:2, PC 18:0-18:2, PC 16:0-18:1, PC 18:0-18:1, PC 16:0-20:4, PC 18:0-20:4 and PC 16:0-22:6 of chemical standards. Data were acquired in MRM mode on a 6500 QTRAP LC-MS.

**Figure S5b. Identity verification of the lipid markers by chemical standards.**

**
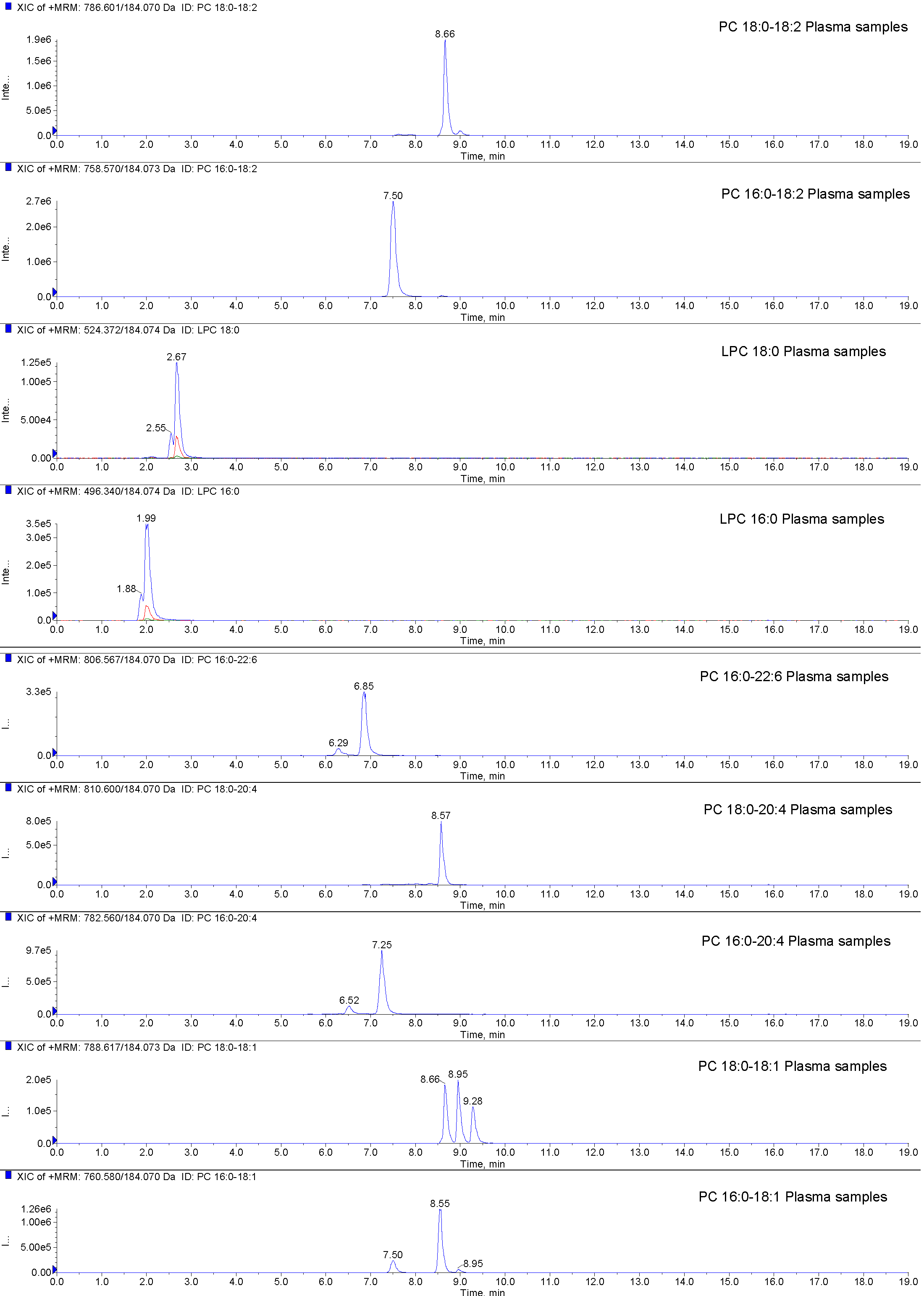
**

**Figure S5b. Identity verification of the lipid markers by chemical standards.**

XIC and retention time of LPC 16:0, LPC 18:0, PC 16:0-18:2, PC 18:0-18:2, PC 16:0-18:1, PC 18:0-18:1, PC 16:0-20:4, PC 18:0-20:4 and PC 16:0-22:6 of plasma samples. Data were acquired in MRM mode on a 6500 QTRAP LC-MS.

**Figure S6a. The enrichment plot of GSEA.**


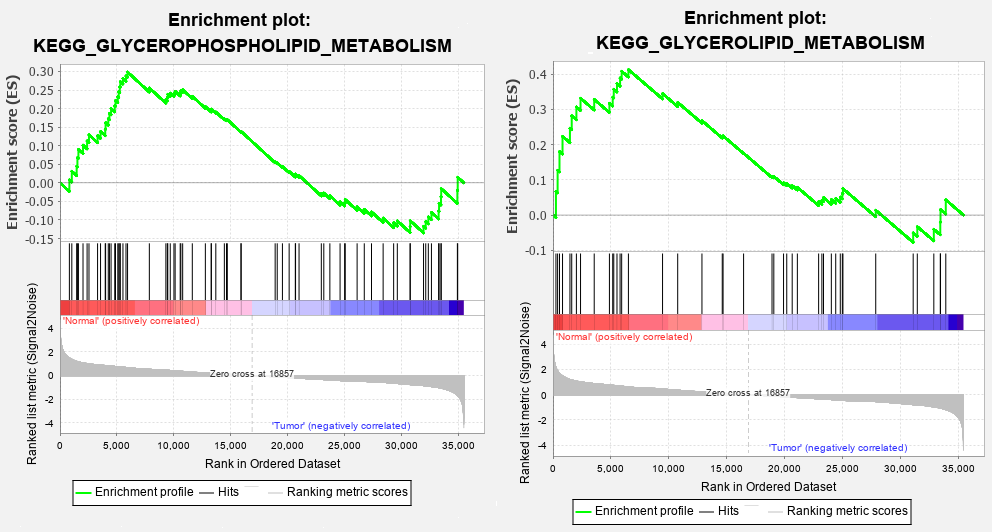


**Figure S6a. The enrichment plot of GSEA.**

The enrichment plot of glycerophospholipid metabolism and glycerolipid metabolism pathway by GSEA algorithm.

**Figure S6b. The heat map presenting the expressive profiles of genes in the pathway of glycerophospholipid metabolism and glycerolipid metabolism.**


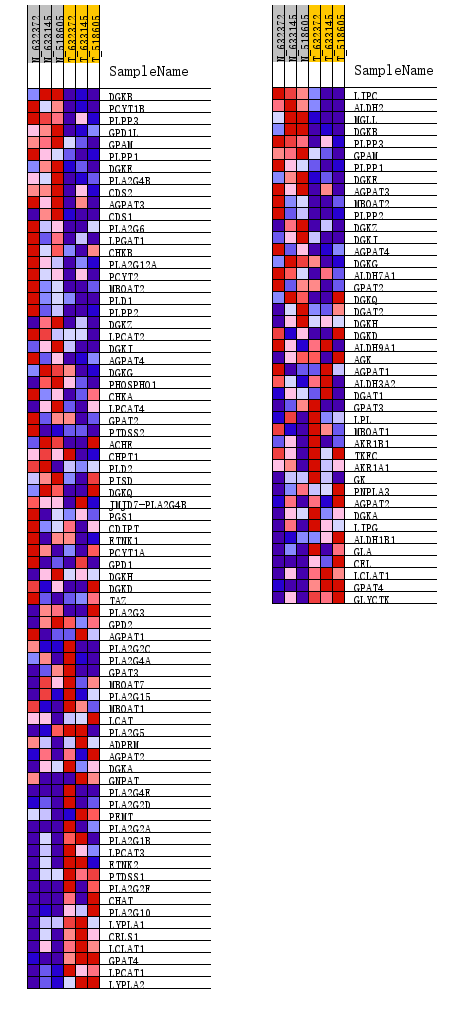


**Table S1. Parameters of the MRM transitions.**

| **Lipid name** | **Q1 m/z in MRM assay** | **Q3 m/z in MRM assay** | **Dwell time** | **DP** | **CE** | **Adduct ion** |
| --- | --- | --- | --- | --- | --- | --- |
| LPC 16:0 | 496.34 | 478.33/258.111/184.074 | 30 ms | 90 | 30 | [M+H]+ |
| LPC 18:0 | 524.37 | 506.361/258.111/184.074 | 30 ms | 90 | 30 | [M+H]+ |
| LPC 18:2 | 520.34 | 502.32/184.074/104.1 | 30 ms | 90 | 30 | [M+H]+ |
| PC 34:1; PC 16:0-18:1 | 760.58 | 184.074 | 30 ms | 90 | 30 | [M+H]+ |
| PC 34:2; PC 16:0-18:2 | 758.57 | 184.074 | 30 ms | 90 | 30 | [M+H]+ |
| PC 36:4; PC 16:0-20:4 | 782.56 | 184.074 | 30 ms | 90 | 30 | [M+H]+ |
| PC 38:6; PC 16:0-22:6 | 806.56 | 184.074 | 30 ms | 90 | 30 | [M+H]+ |
| PC 36:1; PC 18:0-18:1 | 788.62 | 184.074 | 30ms | 90 | 30 | [M+H]+ |
| PC 36:2; PC 18:0-18:2 | 786.6 | 184.074 | 30 ms | 90 | 30 | [M+H]+ |
| PC 38:4; PC 18:0-20:4 | 810.6 | 184.074 | 30 ms | 90 | 30 | [M+H]+ |
| TAG 18:1-18:2-18:3 | 896.76 | 601.48/599.48/597.48 | 30 ms | 90 | 30 | [M+NH4]+ |

**Table S1. Parameters of the MRM transitions.**

Parameters of the transitions representing the 11 target lipids (PC 16:0-18:1, 16:0-18:2, 18:0-18:1, 18:0-18:2 and 16:0-22:6, LPC 13:0, 16:0, 18:0, and 20:4, TG 18:1-18:2-18:3 and TG 8:0-8:0-8:0) in the 19 min LC-MS run in positive ion mode.

**Table S2. GSEA result of the RNA sequencing analysis.**

| NAME | SIZE | ES | NES | NOM p-val | FDR q-val | RANK AT MAX | LEADING EDGE |
| --- | --- | --- | --- | --- | --- | --- | --- |
| KEGG_GLYCEROLIPID_METABOLISM | 43 | 0.41296 | 1.409525 | 0 | 0 | 6512 | tags=37%, list=18%, signal=46% |
| KEGG_GLYCEROPHOSPHOLIPID_METABOLISM | 75 | 0.298389 | 1.221239 | 0 | 0 | 5930 | tags=32%, list=17%, signal=38% |

**Table S2. GSEA result of the RNA sequencing analysis in terms of two marker related KEGG pathways.**

**Table S3a. Expressive profiles of genes in glycerophospholipid metabolism.**

| PROBE | DESCRIPTION<br>(from dataset) | RANK IN GENE LIST | RANK METRIC SCORE (Heathy/tumor) | RUNNING ES |
| --- | --- | --- | --- | --- |
| DGKB | diacylglycerol kinase beta | 823 | 1.478923 | 0.008189 |
| PCYT1B | phosphate cytidylyltransferase 1, choline, beta | 1037 | 1.367433 | 0.031252 |
| PLPP3 | phospholipid phosphatase 3 | 1463 | 1.197441 | 0.044705 |
| GPD1L | glycerol-3-phosphate dehydrogenase 1 like | 1529 | 1.181056 | 0.067988 |
| GPAM | glycerol-3-phosphate acyltransferase, mitochondrial | 1617 | 1.156866 | 0.090135 |
| PLPP1 | phospholipid phosphatase 1 | 2020 | 1.068811 | 0.101503 |
| DGKE | diacylglycerol kinase epsilon | 2378 | 1.013752 | 0.112972 |
| PLA2G4B | phospholipase A2 group IVB | 2544 | 0.988209 | 0.129326 |
| CDS2 | CDP-diacylglycerol synthase 2 | 3298 | 0.899554 | 0.127171 |
| AGPAT3 | 1-acylglycerol-3-phosphate O-acyltransferase 3 | 3559 | 0.875693 | 0.138446 |
| CDS1 | CDP-diacylglycerol synthase 1 | 3970 | 0.849147 | 0.144916 |
| PLA2G6 | phospholipase A2 group VI | 4003 | 0.846141 | 0.162008 |
| LPGAT1 | lysophosphatidylglycerol acyltransferase 1 | 4238 | 0.819966 | 0.172833 |
| CHKB | choline kinase beta | 4339 | 0.809752 | 0.187229 |
| PLA2G12A | phospholipase A2 group XIIA | 4483 | 0.797955 | 0.200158 |
| PCYT2 | phosphate cytidylyltransferase 2, ethanolamine | 4806 | 0.769364 | 0.207419 |
| MBOAT2 | membrane bound O-acyltransferase domain containing 2 | 4872 | 0.763721 | 0.221825 |
| PLD1 | phospholipase D1 | 5074 | 0.745099 | 0.23199 |
| PLPP2 | phospholipid phosphatase 2 | 5157 | 0.739023 | 0.245391 |
| DGKZ | diacylglycerol kinase zeta | 5247 | 0.73133 | 0.25843 |
| LPCAT2 | lysophosphatidylcholine acyltransferase 2 | 5293 | 0.726655 | 0.272613 |
| DGKI | diacylglycerol kinase iota | 5525 | 0.705934 | 0.281097 |
| AGPAT4 | 1-acylglycerol-3-phosphate O-acyltransferase 4 | 5793 | 0.680987 | 0.288033 |
| DGKG | diacylglycerol kinase gamma | 5930 | 0.667656 | 0.298389 |
| PHOSPHO1 | phosphoethanolamine/phosphocholine phosphatase | 7842 | 0.526741 | 0.255567 |
| CHKA | choline kinase alpha | 9294 | 0.455119 | 0.224226 |
| LPCAT4 | lysophosphatidylcholine acyltransferase 4 | 9424 | 0.448126 | 0.23011 |
| GPAT2 | glycerol-3-phosphate acyltransferase 2, mitochondrial | 9460 | 0.444907 | 0.238583 |
| PTDSS2 | phosphatidylserine synthase 2 | 9678 | 0.427387 | 0.241539 |
| ACHE | acetylcholinesterase (Cartwright blood group) | 10001 | 0.403615 | 0.24102 |
| CHPT1 | choline phosphotransferase 1 | 10106 | 0.39693 | 0.246523 |
| PLD2 | phospholipase D2 | 10551 | 0.364311 | 0.241719 |
| PISD | phosphatidylserine decarboxylase | 10607 | 0.360624 | 0.247834 |
| DGKQ | diacylglycerol kinase theta | 10768 | 0.349324 | 0.250741 |
| JMJD7-PLA2G4B | JMJD7-PLA2G4B readthrough | 11613 | 0.291562 | 0.233082 |
| PGS1 | phosphatidylglycerophosphate synthase 1 | 12759 | 0.220296 | 0.205397 |
| CDIPT | CDP-diacylglycerol--inositol 3-phosphatidyltransferase | 13276 | 0.185584 | 0.194756 |
| ETNK1 | ethanolamine kinase 1 | 13281 | 0.185406 | 0.198587 |
| PCYT1A | phosphate cytidylyltransferase 1, choline, alpha | 13677 | 0.161112 | 0.190847 |
| GPD1 | glycerol-3-phosphate dehydrogenase 1 | 14426 | 0.132826 | 0.172525 |
| DGKH | diacylglycerol kinase eta | 14615 | 0.120843 | 0.16978 |
| DGKD | diacylglycerol kinase delta | 14686 | 0.116234 | 0.170274 |
| TAZ | tafazzin | 15889 | 0.056738 | 0.137499 |
| PLA2G3 | phospholipase A2 group III | 15897 | 0.056502 | 0.138503 |
| GPD2 | glycerol-3-phosphate dehydrogenase 2 | 18886 | -0.10831 | 0.056333 |
| AGPAT1 | 1-acylglycerol-3-phosphate O-acyltransferase 1 | 19068 | -0.11926 | 0.053752 |
| PLA2G2C | phospholipase A2 group IIC | 19524 | -0.14117 | 0.043892 |
| PLA2G4A | phospholipase A2 group IVA | 20114 | -0.17116 | 0.030881 |
| GPAT3 | glycerol-3-phosphate acyltransferase 3 | 20655 | -0.20493 | 0.019973 |
| MBOAT7 | membrane bound O-acyltransferase domain containing 7 | 20677 | -0.20575 | 0.023755 |
| PLA2G15 | phospholipase A2 group XV | 20983 | -0.2235 | 0.019887 |
| MBOAT1 | membrane bound O-acyltransferase domain containing 1 | 22938 | -0.3241 | -0.02846 |
| LCAT | lecithin-cholesterol acyltransferase | 23156 | -0.33647 | -0.02744 |
| PLA2G5 | phospholipase A2 group V | 23688 | -0.36491 | -0.03469 |
| ADPRM | ADP-ribose/CDP-alcohol diphosphatase, manganese dependent | 24584 | -0.4186 | -0.05109 |
| AGPAT2 | 1-acylglycerol-3-phosphate O-acyltransferase 2 | 24985 | -0.44403 | -0.05295 |
| DGKA | diacylglycerol kinase alpha | 25036 | -0.44774 | -0.04484 |
| GNPAT | glyceronephosphate O-acyltransferase | 26080 | -0.47633 | -0.0642 |
| PLA2G4E | phospholipase A2 group IVE | 26709 | -0.50193 | -0.07128 |
| PLA2G2D | phospholipase A2 group IID | 27343 | -0.52022 | -0.07811 |
| PEMT | phosphatidylethanolamine N-methyltransferase | 28364 | -0.56927 | -0.09484 |
| PLA2G2A | phospholipase A2 group IIA | 29287 | -0.63858 | -0.10732 |
| PLA2G1B | phospholipase A2 group IB | 29609 | -0.66589 | -0.10223 |
| LPCAT3 | lysophosphatidylcholine acyltransferase 3 | 30733 | -0.75904 | -0.11784 |
| ETNK2 | ethanolamine kinase 2 | 30743 | -0.7598 | -0.10193 |
| PTDSS1 | phosphatidylserine synthase 1 | 31911 | -0.8439 | -0.11697 |
| PLA2G2F | phospholipase A2 group IIF | 32104 | -0.86029 | -0.1041 |
| CHAT | choline O-acetyltransferase | 32331 | -0.89145 | -0.09153 |
| PLA2G10 | phospholipase A2 group X | 32604 | -0.92648 | -0.07952 |
| LYPLA1 | lysophospholipase 1 | 33235 | -1.01696 | -0.0757 |
| CRLS1 | cardiolipin synthase 1 | 33275 | -1.02296 | -0.05504 |
| LCLAT1 | lysocardiolipin acyltransferase 1 | 33443 | -1.05258 | -0.03737 |
| GPAT4 | glycerol-3-phosphate acyltransferase 4 | 33463 | -1.05713 | -0.01543 |
| LPCAT1 | lysophosphatidylcholine acyltransferase 1 | 34881 | -1.66953 | -0.01998 |
| LYPLA2 | lysophospholipase 2 | 34910 | -1.68889 | 0.015153 |

**Table S3b. Expressive profiles of genes in glycerolipid metabolism.**

| PROBE | DESCRIPTION<br>(from dataset) | RANK IN GENE LIST | RANK METRIC SCORE (Healthy/tumor) | RUNNING ES |
| --- | --- | --- | --- | --- |
| LIPC | lipase C, hepatic type | 254 | 2.16732 | 0.06703 |
| ALDH2 | aldehyde dehydrogenase 2 family member | 399 | 1.874178 | 0.12713 |
| MGLL | monoglyceride lipase | 561 | 1.686729 | 0.180332 |
| DGKB | diacylglycerol kinase beta | 823 | 1.478923 | 0.223595 |
| PLPP3 | phospholipid phosphatase 3 | 1463 | 1.197441 | 0.246543 |
| GPAM | glycerol-3-phosphate acyltransferase, mitochondrial | 1617 | 1.156866 | 0.28183 |
| PLPP1 | phospholipid phosphatase 1 | 2020 | 1.068811 | 0.307069 |
| DGKE | diacylglycerol kinase epsilon | 2378 | 1.013752 | 0.331694 |
| AGPAT3 | 1-acylglycerol-3-phosphate O-acyltransferase 3 | 3559 | 0.875693 | 0.328346 |
| MBOAT2 | membrane bound O-acyltransferase domain containing 2 | 4872 | 0.763721 | 0.317436 |
| PLPP2 | phospholipid phosphatase 2 | 5157 | 0.739023 | 0.334717 |
| DGKZ | diacylglycerol kinase zeta | 5247 | 0.73133 | 0.357242 |
| DGKI | diacylglycerol kinase iota | 5525 | 0.705934 | 0.373588 |
| AGPAT4 | 1-acylglycerol-3-phosphate O-acyltransferase 4 | 5793 | 0.680987 | 0.389361 |
| DGKG | diacylglycerol kinase gamma | 5930 | 0.667656 | 0.408379 |
| ALDH7A1 | aldehyde dehydrogenase 7 family member A1 | 6512 | 0.613126 | 0.41296 |
| GPAT2 | glycerol-3-phosphate acyltransferase 2, mitochondrial | 9460 | 0.444907 | 0.344954 |
| DGKQ | diacylglycerol kinase theta | 10768 | 0.349324 | 0.319997 |
| DGAT2 | diacylglycerol O-acyltransferase 2 | 12862 | 0.213548 | 0.268191 |
| DGKH | diacylglycerol kinase eta | 14615 | 0.120843 | 0.222842 |
| DGKD | diacylglycerol kinase delta | 14686 | 0.116234 | 0.224845 |
| ALDH9A1 | aldehyde dehydrogenase 9 family member A1 | 16458 | 0.022443 | 0.175591 |
| AGK | acylglycerol kinase | 18913 | -0.10994 | 0.11004 |
| AGPAT1 | 1-acylglycerol-3-phosphate O-acyltransferase 1 | 19068 | -0.11926 | 0.109774 |
| ALDH3A2 | aldehyde dehydrogenase 3 family member A2 | 19908 | -0.16399 | 0.09169 |
| DGAT1 | diacylglycerol O-acyltransferase 1 | 20185 | -0.17606 | 0.089922 |
| GPAT3 | glycerol-3-phosphate acyltransferase 3 | 20655 | -0.20493 | 0.083692 |
| LPL | lipoprotein lipase | 21101 | -0.22627 | 0.07887 |
| MBOAT1 | membrane bound O-acyltransferase domain containing 1 | 22938 | -0.3241 | 0.038107 |
| AKR1B1 | aldo-keto reductase family 1 member B | 23218 | -0.33987 | 0.041863 |
| TKFC | triokinase and FMN cyclase | 23359 | -0.34694 | 0.049787 |
| AKR1A1 | aldo-keto reductase family 1 member A1 | 24035 | -0.38593 | 0.043935 |
| GK | glycerol kinase | 24414 | -0.40861 | 0.047248 |
| PNPLA3 | patatin like phospholipase domain containing 3 | 24808 | -0.4329 | 0.050969 |
| AGPAT2 | 1-acylglycerol-3-phosphate O-acyltransferase 2 | 24985 | -0.44403 | 0.061201 |
| DGKA | diacylglycerol kinase alpha | 25036 | -0.44774 | 0.075118 |
| LIPG | lipase G, endothelial type | 27869 | -0.54373 | 0.013743 |
| ALDH1B1 | aldehyde dehydrogenase 1 family member B1 | 31103 | -0.78695 | -0.05063 |
| GLA | galactosidase alpha | 31458 | -0.80665 | -0.03301 |
| CEL | carboxyl ester lipase | 32885 | -0.96274 | -0.04033 |
| LCLAT1 | lysocardiolipin acyltransferase 1 | 33443 | -1.05258 | -0.02002 |
| GPAT4 | glycerol-3-phosphate acyltransferase 4 | 33463 | -1.05713 | 0.015634 |
| GLYCTK | glycerate kinase | 33919 | -1.17848 | 0.043131 |
